# Single-nucleus transcriptomic data of susceptible and resistant tobacco stems after black shank infection

**DOI:** 10.1016/j.dib.2026.113095

**Published:** 2026-07-23

**Authors:** Xiaolin Lu, Zhijun Tong, Canhua Lu, Chen Tan, Hongyu Chen, Longjiang Fan, Bingguang Xiao

**Affiliations:** a Key Laboratory of Tobacco Biotechnological Breeding, National Tobacco Genetic Engineering Research Center, Yunnan Academy of Tobacco Agricultural Sciences, Kunming 650021, China; b Institute of Crop Sciences & Institute of Bioinformatics, Zhejiang University, Hangzhou 310058, China

**Keywords:** *Phytophthora nicotianae*, Tobacco, snRNA-seq, Plant–pathogen interaction, Disease resistance

## Abstract

Black shank, caused by *Phytophthora nicotianae*, is a major disease in tobacco production. Previous transcriptomic work has used *Nicotiana tabacum* L. cv. Honghuadajinyuan (HD) as a susceptible tobacco material in black shank research. Presented here is a single-nucleus RNA sequencing (snRNA-seq) dataset generated from stem tissues of two tobacco accessions with contrasting responses to black shank, namely the susceptible cultivar HD and the black shank-resistant near-isogenic line HDR7 derived from HD. Stem tissues surrounding the inoculation site were collected at 48 h post inoculation with *P. nicotianae* race 0 and used for nucleus isolation, library construction, and sequencing. Because plant tissues are often difficult to dissociate into intact single cells, nucleus-based transcriptomic profiling provides a practical way to generate cell-resolved expression data from these samples. The dataset contains snRNA profiles from infected tobacco stems of both susceptible and resistant materials and can be reused for cell-type annotation, marker screening, comparative analysis of transcriptional features between accessions, and integration with future tobacco stem or disease-related single-cell and single-nucleus transcriptomic datasets.

Specifications Table

**Table:** 

Subject	Biology
Specific subject area	Single-nucleus transcriptome sequencing (snRNA-seq), Plant pathology, *Phytophthora nicotianae*.
Type of data	Figures, tables, graphs, and processed, filtered, and analyzed data.
Data collection	Nuclei were isolated from infected tobacco stem tissues in Galbraith buffer supplemented with DTT and RNase inhibitor, stained with DAPI, and sorted using a BD FACSAria III flow cytometer. Single-nucleus libraries were prepared on the 10x Genomics Chromium Single Cell 3′ platform, and sequenced on an Illumina NovaSeq 6000 instrument. Raw reads were processed with Cell Ranger, followed by quality filtering, clustering, and marker gene analysis.
Data source location	Tobacco stem samples were collected at the Key Laboratory of Tobacco Biotechnological Breeding, National Tobacco Genetic Engineering Research Center, Yunnan Academy of Tobacco Agricultural Sciences, Kunming, Yunnan 650,021, China. Subsequent sequencing data processing and bioinformatic analysis were carried out at the Institute of Crop Sciences & Institute of Bioinformatics, Zhejiang University, Hangzhou 310,058, China.
Data accessibility	Repository name: Genome Sequence Archive (GSA) Data identification number: CRA040424 Direct URL to data: https://ngdc.cncb.ac.cn/gsa/search?searchTerm=CRA040424
Related research article	None.

## 1 Value of the Data

• These snRNA-seq data provide cell-type-resolved transcriptomic profiles from tobacco stem tissues of the susceptible cultivar Honghuadajinyuan (HD) and the black shank-resistant near-isogenic line HDR7 at 48 h post inoculation with *Phytophthora nicotianae* race 0.
• The dataset documents cellular heterogeneity and gene expression patterns in infected tobacco stems during an early stage of black shank infection.
• The dataset can be reused to identify marker genes, compare cell populations between susceptible and resistant tobacco cultivars, and examine transcriptional features associated with black shank infection.
• The dataset may be useful to researchers working on tobacco disease resistance, plant–pathogen interactions, and single-cell or single-nucleus transcriptomics in plants.
• The dataset can also be used as a reference resource for comparative analyses with transcriptomic data from other tobacco tissues, infection stages, or stress-related conditions.

## 2 Background

Black shank, caused by *Phytophthora nicotianae*, is one of the most important diseases of cultivated tobacco worldwide. The pathogen can infect roots, stems, and leaves during plant growth, and previous transcriptomic analyses have been used to examine tobacco responses to *P. nicotianae* race 0, including analyses of stem tissues after inoculation [1,2]. Honghuadajinyuan (HD) has also been used as a susceptible tobacco material in black shank research [1,2]. To extend available transcriptomic resources for tobacco black shank research at higher cellular resolution, a single-nucleus RNA sequencing dataset was generated from stem tissues collected around the inoculation site at 48 h post inoculation with *P. nicotianae* race 0. The dataset includes two tobacco accessions with contrasting disease responses, namely HD and HDR7, a black shank-resistant near-isogenic line derived from HD. Single-nucleus RNA sequencing (snRNA-seq) was selected because nucleus-based approaches are suitable for plant tissues that are difficult to dissociate into intact single cells due to rigid cell walls and tissue complexity [3].

## 3 Data Description

This dataset contains snRNA-seq data generated from tobacco stem tissues collected at 48 h post inoculation with *P. nicotianae* race 0 from two tobacco materials with contrasting responses to black shank, namely HD_48h and HDR7_48h. Basic sequencing and data quality metrics for the two libraries are summarized in Table 1. HD_48h contained 17,548 estimated cells, with 23,927 mean reads per cell and 1277 median genes per cell, whereas HDR7_48h contained 11,261 estimated cells, with 38,144 mean reads per cell and 2070 median genes per cell. The Q30 values of RNA reads were 93.2% for HD_48h and 92.6% for HDR7_48h, and the fractions of reads in cells were 78.5% and 75.9%. A total of 49,752 genes were detected in HD_48h, compared with 48,806 genes in HDR7_48h.

**Table 1:** Summary of two snRNA-seq datasets from black shank-infected tobacco stems and sequencing metrics. HD, a black shank-susceptible tobacco accession, and HDR7, a black shank-resistant near-isogenic line derived from HD.

Sample ID	HD_48h	HDR7_48h
Disease response	Susceptible	Resistant
Estimated Number of Cells	17,548	11,261
Mean Reads per Cell	23,927	38,144
Median Genes per Cell	1277	2070
Q30 Bases in RNA Read	93.2%	92.6%
Fraction Reads in Cells	78.5%	75.9%
Total Genes Detected	49,752	48,806

**Fig. 1:**
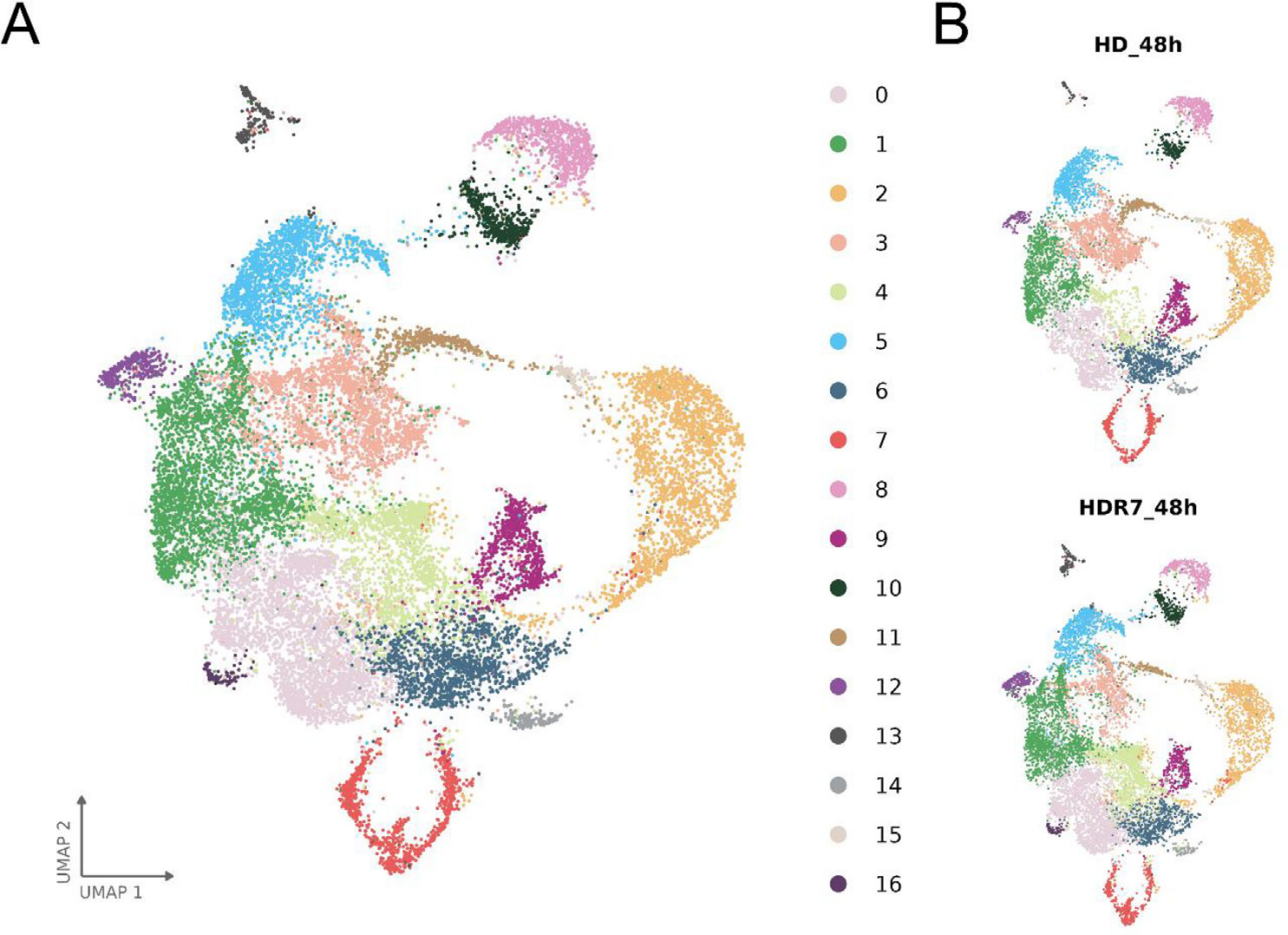
UMAP visualization of the tobacco stem snRNA-seq dataset. (A) UMAP plot of the integrated dataset of two samples colored by Seurat cluster identity. (B) Sample-specific UMAP plots showing the distributions of cells from HD_48h and HDR7_48h using the same cluster color scheme as in panel A.

An overview of the integrated dataset is shown in Fig. 1A, in which cells from both samples are displayed on a shared UMAP embedding and colored by Seurat cluster identity. Sample-specific UMAP plots for HD_48h and HDR7_48h are shown in Fig. 1B, allowing the two libraries to be viewed separately using the same cluster color scheme.

**Fig. 2:**
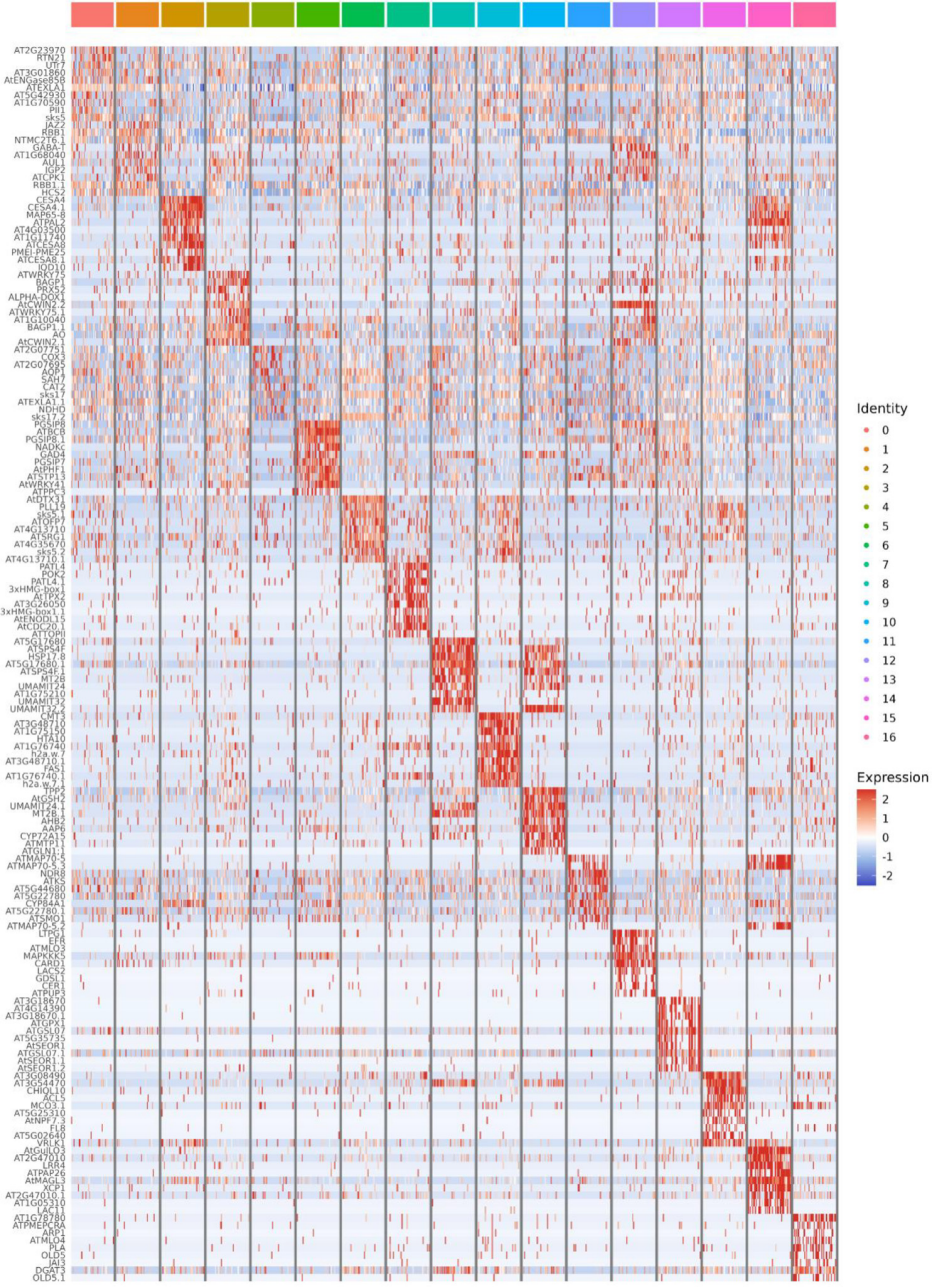
Heatmap of representative marker genes across different clusters based on the integrated tobacco snRNA-seq dataset.

Cluster-level marker expression based on the integrated dataset (Fig. 1A) is shown in Fig. 2, which presents a heatmap of selected marker genes across different clusters. In this figure, cells are grouped by Seurat cluster identity, and rows represent representative marker genes selected from the cluster marker table generated during downstream analysis.

Cells are grouped by Seurat cluster identity, and rows represent selected marker genes from the cluster marker table. The color scale indicates scaled expression levels.

### 3.1 Cell-type annotation and putative infection-related roles

Previous single-cell transcriptomic studies have shown that plant responses to pathogen infection are heterogeneous across cell types, and that cell-type-resolved analysis can reveal infection-associated transcriptional features that may be obscured in bulk tissue-level analyses [4,5]. Therefore, annotating stem cell populations in this dataset provides a cellular framework for describing tobacco responses to *P. nicotianae* infection at cell-type resolution. Based on the integrated snRNA-seq dataset, ten major stem cell classes were annotated, including Parenchyma cell, Phloem, Xylem, Immune-activated cell, G2/M phase cell, Companion cell, S phase cell, Epidermis, Sieve element, and Procambium.

**Fig. 3:**
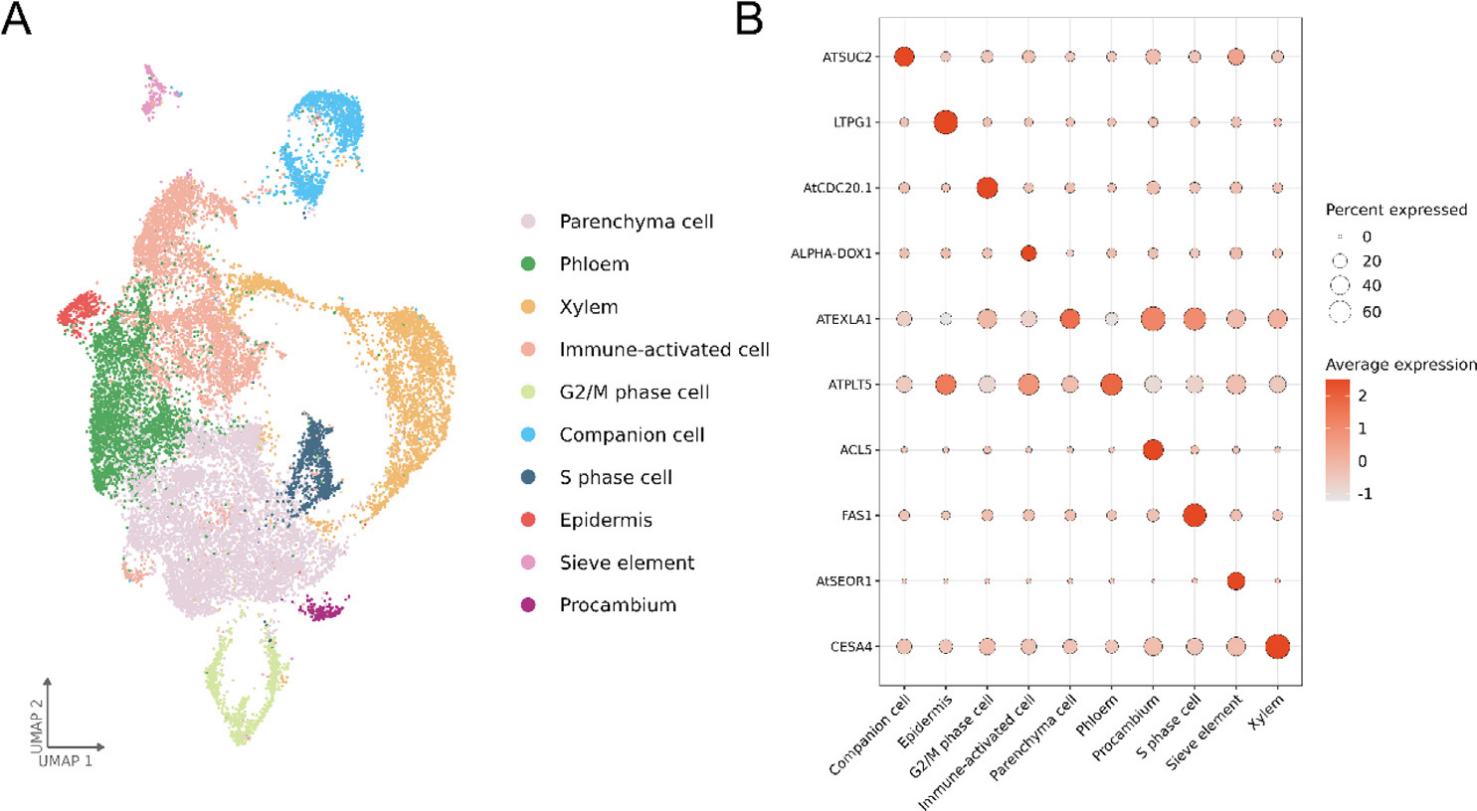
Cell-type annotation and representative marker gene expression in the tobacco stem based on the snRNA-seq dataset. (A) UMAP plot colored by the final cell-type annotation labels. (B) Dot plot showing representative marker genes for the annotated cell classes. Dot size indicates the percentage of cells expressing each gene within each annotated cell class, and color indicates scaled average expression.

The annotation result is shown in Fig. 3A, in which the UMAP embedding is colored by the final annotation labels. A dot plot summarizing representative marker genes for the annotated cell classes is shown in Fig. 3B In the dot plot, dot size represents the percentage of cells expressing each gene within each annotated cell class, and color represents scaled average expression.

To further address the potential contribution of different stem cell types during black shank infection, we summarized the putative infection-related roles of the 1 types in Table 2. Epidermal cells may contribute to cuticular barrier formation, pathogen perception, and early defense signaling, whereas xylem cells may be associated with secondary cell wall formation, lignification, and vascular reinforcement. Phloem-associated cells, including phloem, companion cells, and sieve elements, may participate in transport, metabolic redistribution, systemic signaling, callose-associated sieve-tube sealing, and redox regulation. The immune-activated population was interpreted as a defense-responsive transcriptional state rather than a conventional anatomical cell type. S phase and G2/M phase cells represented proliferating cell states, which may reflect stem growth and potential infection-associated tissue renewal.

**Table 2:** Putative roles of annotated tobacco stem cell types during black shank infection.

Cell type	Supporting genes	Putative infection-related role
Companion cell	*UMAMIT32, UMAMIT24, ATSPS4F, TPP2, HSP17.8, MT2B*	Long-distance transport, metabolic support, and stress-related source–sink coordination
Epidermis	*LTPG1, EFR, MAPKKK5, LACS2, CER1, CER8, ATSERK2, AtPERK4*	Cuticular barrier, pathogen perception, and early defense signaling
G2/M phase cell	*POK2, AtTPX2, AtCDC20.1, ATTOPII, PAKRP2, ATSYP111*	Mitotic cell state potentially associated with growth or infection-related tissue renewal
Immune-activated cell	*AtWRKY75, AtWRKY41, NADKc, AO, ABCG36, ATPAL1, ATGSTU8, CRK10*	Defense-responsive transcriptional state associated with WRKY regulation, redox balance, and phenylpropanoid metabolism
Parenchyma cell	*BGLU17, AtBGAL10, AtDTX31, APY2, LHY, ATSRG1*	Basal metabolic support, cell-wall remodeling, and local stress response
Phloem	*JAZ2, GABA-T, ATCPK1, ATPLT5, ATWRKY40, ATNR2, AIM1*	Hormone/Ca2+ signaling, transport, and systemic defense-related redistribution
Procambium	*ACL5, MCO3, AtNPF7.3, VRLK1, ATPLC2, ANAC037, ATGRP7*	Vascular development, tissue remodeling, and stress-associated regulation
S phase cell	*CMT3, HTA10, H2A.W.7, FAS1, AtJMJ17, G-H2AX, KYP*	DNA replication and chromatin assembly, representing a proliferating cell state
Sieve element	*ATGSL07, AtSEOR1, ATGPX1, ATGPX3, ATMLO13, AtCLO4*	Phloem transport, callose-associated sieve-tube sealing, and redox regulation
Xylem	*CESA4, ATCESA7, ATCESA8, IRX9, ATPAL2, ATCCR1, CYP84A1, CYP98A3*	Secondary cell wall formation, lignification, and vascular reinforcement

### 3.2 Functional enrichment of HD- and HDR7-biased genes across cell types

**Fig. 4:**
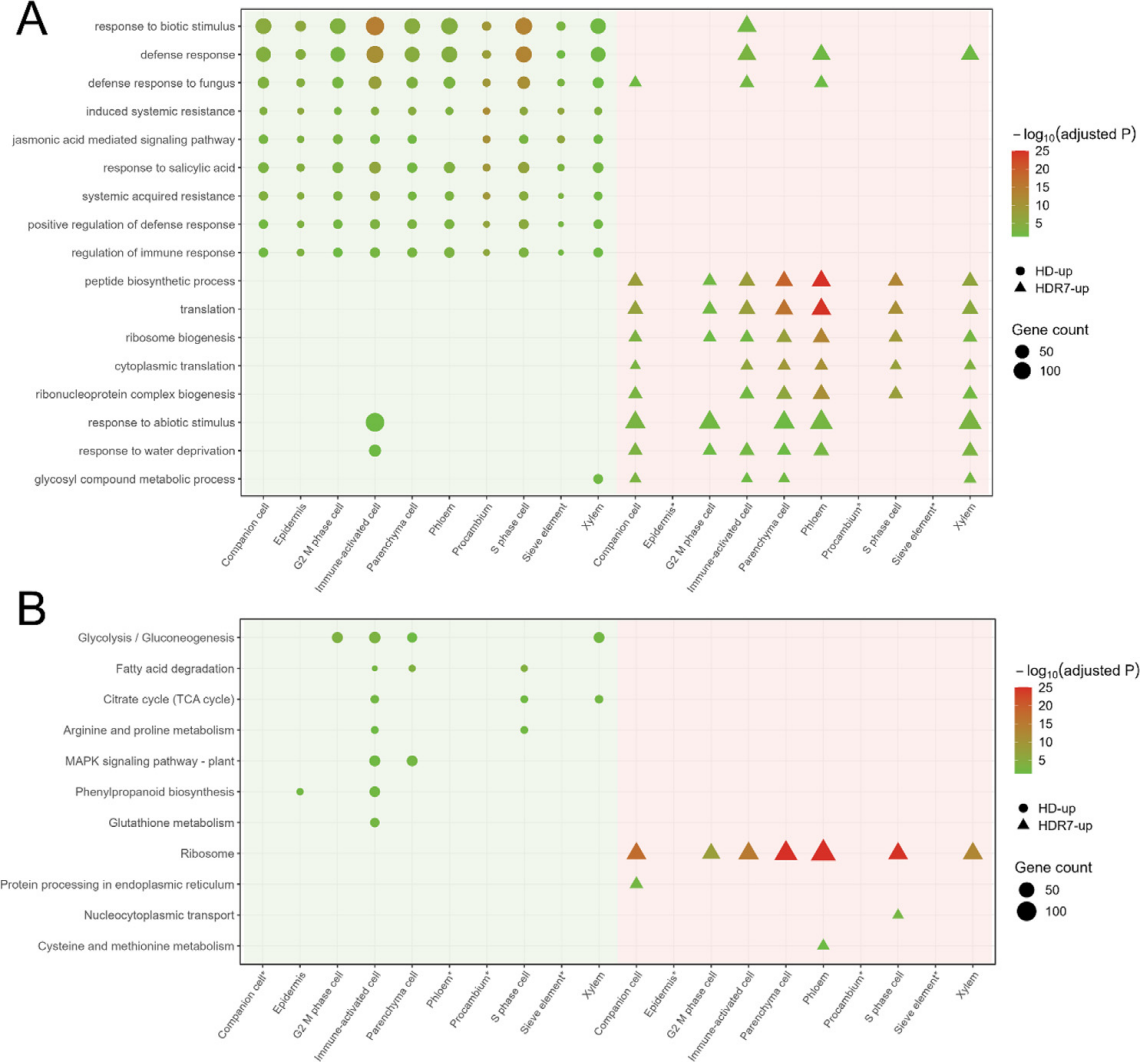
Functional enrichment of HD- and HDR7-biased genes across annotated tobacco stem cell types. (A) Selected GO Biological Process terms enriched in HD-upregulated and HDR7-upregulated genes across annotated cell types. (B) Selected KEGG pathways enriched in HD-upregulated and HDR7-upregulated genes across annotated cell types.

After cell-type annotation, we further summarized the functional categories represented by HD- and HDR7-biased genes within each annotated cell type. This analysis was performed to compare transcriptional features between HD and HDR7 at cell-type resolution, extending the comparison beyond whole-sample or bulk-level expression patterns. The representative GO and KEGG terms shown in Fig. 4 provide an overview of the functional modules captured by the dataset across different tobacco stem cell types.

The enrichment patterns showed that HD-upregulated genes were broadly associated with defense- and stress-related categories, whereas HDR7-upregulated genes were represented by a more restricted set of functional categories, including translation-, ribosome-, protein-processing-, and transport-related processes. In HD, the broad enrichment of immune- and stress-related terms may be related to stronger infection pressure, more extensive cellular damage, or activation of broad stress responses at 48 h post inoculation. In HDR7, the enrichment of translation- and protein-processing-related categories may reflect a comparatively maintained cellular activity and a more focused transcriptional adjustment under infection. These results provide a cell-type-resolved functional overview of HD- and HDR7-biased responses during tobacco black shank infection and offer a reference resource for comparative studies of tobacco responses to *P. nicotianae* infection.

In both panels, the left block represents HD-upregulated gene sets and the right block represents HDR7-upregulated gene sets. Dot size indicates the number of genes associated with each term, and color indicates −log10(adjusted P). Circles and triangles represent HD-upregulated and HDR7-upregulated gene sets, respectively. Asterisks next to cell-type labels indicate cell types without significant enrichment among the selected representative terms.

## 4 Experimental Design, Materials and Methods

Two tobacco accessions with contrasting black shank responses were included in the dataset: *Nicotiana tabacum* L. cv. Honghuadajinyuan (HD), a susceptible flue-cured tobacco cultivar, and HDR7, a black shank-resistant near-isogenic line derived from HD. HDR7 was developed by the National Tobacco Genetic Engineering Research Center using HD as the recurrent parent and RBST, a previously reported black shank-resistant breeding line, as the resistance donor through nine generations of backcrossing followed by selfing [1,6]. RBST carries the black shank resistance gene pH, which originated from the wild tobacco species *Nicotiana plumbaginifolia* and confers high resistance to race 0 of the black shank pathogen [7]. Linked molecular markers were used during backcrossing and selfing to select plants carrying the pH gene and to confirm homozygosity in HDR7. Representative disease symptoms of RBST, HDR7, and HD at 14 days after inoculation with *P. nicotianae* race 0 are provided in Supplementary Fig. S1 to show the contrasting black shank responses of the materials.

Plants were inoculated with *P. nicotianae* race 0 at the stem inoculation site. Briefly, nine small wounds arranged in three rows were made using a sterile toothpick, and the inoculated region was wrapped with sterile absorbent cotton. Stem tissues approximately 1 cm above and below the inoculation site were collected at 48 h post inoculation, rapidly frozen, and stored at −80 °C until subsequent processing.

For snRNA sequencing, frozen stem tissues were ground in liquid nitrogen, and nuclei were isolated in Galbraith buffer supplemented with DTT and RNase inhibitor. After filtration and centrifugation, nuclei were stained with DAPI and sorted on a BD FACSAria III. Nuclear integrity and concentration were assessed before library construction. Single-nucleus libraries were generated using the 10x Genomics Chromium Single Cell 3′ v4 platform and sequenced on an Illumina NovaSeq 6000 instrument.

Sequencing data were processed with Cell Ranger (9.0.1), and filtered feature-barcode matrices from HDR7_48h and HD_48h were imported into Seurat in R for downstream analysis. Seurat objects were created using min.cells = 3 and min.features = 200, and additional filtering retained nuclei with nFeature_RNA < 10,000 and nCount_RNA < 25,000. The two datasets were integrated using the SCTransform-based Seurat workflow. Briefly, each dataset was normalized with SCTransform, 7000 integration features were selected, integration anchors were identified using RPCA with HDR7_48h as the reference, and the integrated object was generated using IntegrateData. Principal component analysis was performed using 50 principal components, followed by neighbor graph construction, graph-based clustering at a resolution of 0.5, and UMAP visualization.

Differential expression analysis was performed within each annotated cell type to compare HD_48h and HDR7_48h. For each cell type, nuclei assigned to HD_48h and HDR7_48h were extracted from the integrated Seurat object, and differential expression analysis was performed using the FindMarkers function in Seurat with the MAST test, min.pct = 0.1, and logfc.threshold = 0.25. Genes with higher expression in HDR7_48h than in HD_48h were defined as HDR7-upregulated genes, whereas genes with higher expression in HD_48h than in HDR7_48h were defined as HD-upregulated genes. Differentially expressed genes with adjusted *P* value < 0.05 were retained for downstream functional enrichment analysis.

Functional enrichment analysis of HD- and HDR7-upregulated genes was performed using clusterProfiler based on custom GO and KEGG annotation databases constructed from eggNOG-mapper annotation results [8]. Background genes were defined as genes detected in the Seurat object and present in the corresponding custom annotation database. Complete enrichment tables were generated for HD-upregulated and HDR7-upregulated genes in each annotated cell type, and representative significant terms were selected for visualization.

Downstream analyses were performed in R version 4.3.3 [9] using Seurat 4.4.0 [10], ggplot2 3.5.0 [11], reshape2 1.4.4 [12], RColorBrewer 1.1.3 [13], tidyverse 2.0.0 [14], readxl 1.4.3 [15], openxlsx 4.2.7.1 [16], clusterProfiler 4.14.6 [17], and AnnotationDbi 1.68.0 [18].

## Limitations

This dataset is limited by the relatively small sampling size.

## Ethics Statement

The authors have read and complied with the ethical requirements for publication in *Data in Brief*. The current work does not involve human subjects, animal experiments, or data collected from social media platforms.

## CRediT Author Statement

**Xiaolin Lu:** Formal analysis, Visualization, Writing - Original Draft. **Zhijun Tong:** Methodology, Project administration. **Canhua Lu:** Investigation, Resources. **Chen Tan:** Data curation, Formal analysis. **Hongyu Chen:** Supervision, Writing - Review & Editing. **Longjiang Fan:** Supervision, Writing - Review & Editing. **Bingguang Xiao:** Conceptualization, Resources, Supervision.

## Data Availability

GSASingle-nucleus transcriptome dataset of tobacco stem tissues after black shank infection (Original data).

## Appendix Supplementary materials

Image, application 1
