# Supplementary material for: Single-nucleus transcriptomic data of susceptible and resistant tobacco stems after black shank infection
Source: Data Brief. 2026 Jul 23;68:113095. doi: 10.1016/j.dib.2026.113095 (PMC13474382; doi:10.1016/j.dib.2026.113095)
Supplement: Supplementary file 1 [file mmc1.docx]

Supplementary Fig. S1. Disease symptoms of tobacco materials at 14 days after inoculation with *Phytophthora nicotianae* race 0.

Representative photograph showing tobacco plants at 14 days after inoculation with mycelial agar plugs of *P. nicotianae* race 0. From left to right, the trays correspond to RBST, HDR7, HDR7, and HD. RBST and HD were used as the resistant and susceptible parental/control materials, respectively. HDR7 was the black shank-resistant near-isogenic line used in this dataset. RH01 is the internal breeding code for HDR7.
